# Guided CdTe Nanowires Integrated into Fast Near-Infrared Photodetectors

**DOI:** 10.1021/acsami.3c15797

**Published:** 2024-01-04

**Authors:** Yarden Danieli, Ella Sanders, Olga Brontvein, Ernesto Joselevich

**Affiliations:** † Department of Molecular Chemistry and Materials Sciences, Weizmann Institute of Science, Rehovot 76100, Israel; ‡ Chemical Research Support, Weizmann Institute of Science, Rehovot 76100, Israel

**Keywords:** CdTe, infrared, MCT, photodetectors, nanowires

## Abstract

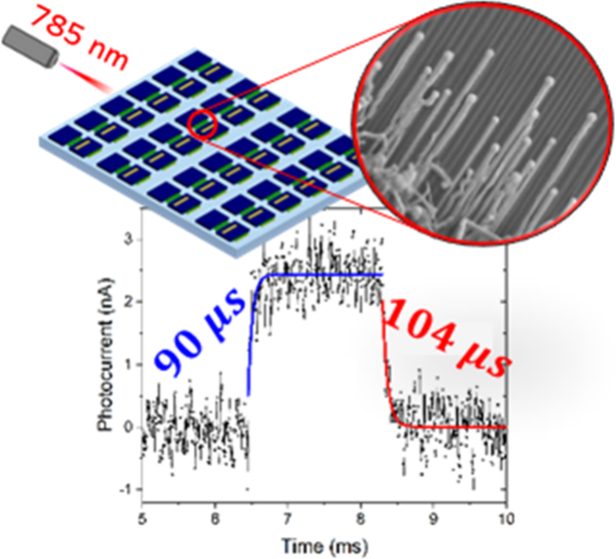

Infrared photodetectors are essential devices for telecommunication and night vision technologies. Two frequently used materials groups for this technology are III–V and II–VI semiconductors, notably, mercury-cadmium-telluride alloys (MCT). However, growing them usually requires expensive substrates that can only be provided on small scales, and their large-scale production as crystalline nanostructures is challenging. In this paper, we present a two-stage process for creating aligned MCT nanowires (NWs). First, we report the growth of planar CdTe nanowires with controlled orientations on flat and faceted sapphire substrates via the vapor–liquid–solid (VLS) mechanism. We utilize this guided growth approach to parallelly integrate the NWs into fast near-infrared photodetectors with characteristic rise and fall times of ∼100 μs at room temperature. An epitaxial effect of the planar growth and the unique structure of the NWs, including size and composition, are suggested to explain the high performance of the devices. In the second stage, we show that cation exchange with mercury can be applied, resulting in a band gap narrowing of up to 55 meV, corresponding to an exchange of 2% Cd with Hg. This work opens new opportunities for creating small, fast, and sensitive infrared detectors with an engineered band gap operating at room temperature.

## 1 Introduction

Semiconductor nanowires (NWs) have been the focus of both fundamental and applied sciences due to their unique physical properties,^1^ as well as being promising components in functional nanodevices such as field-effect transistors (FETs),^2^ light-emitting diodes (LEDs),^3^ photovoltaic cells (PVs),^4^ and photodetectors (PDs).^5^ Efficient scale-up of such functional systems requires precise control over the position and orientation of NW arrays, and several methods were suggested for this purpose.^6−9^ Although these methods achieve the alignment of the NWs, they suffer from severe disadvantages, such as chemical contamination and inaccurate control over the position and polarity. One advantageous alternative came with the emergence of the guided growth approach first presented by Nikoobakht et al.^10^ and extensively expanded by Joselevich and co-workers^11−17^ in which controlled position and orientation of planar NWs are achieved in a single growth step by planar growth, guided by the substrate. Three modes were suggested to control the direction of the NWs growing on the substrate during the past decade. The first one is epitaxy, where lattice mismatch minimization drives guided growth. The second is graphoepitaxy on spontaneously created faceted surfaces where surface energy minimization drives the guided growth.^17^ An additional mode demonstrated lately uses artificial epitaxy, i.e., artificial creation of nanometric patterns on amorphous substrates to guide the growth of semiconductor NWs on top of SiO_2_^18^ and glass.^19^ Guided growth is, in fact, a unique case of in-plane NW growth, which differs dramatically from the common vertical NWs, in both growth conditions and the creation mechanism, as was demonstrated by Rothman et al.^20^ It was also shown by Schvartzman et al.^21^ that high control over the catalyst position, combined with guided growth approach, can yield self-integrating NWs into patterns needed for a specific circuit, further highlighting the technological impact of guided semiconductor NWs. The guided growth technological potential is further manifested by the increasing list of reports about the growth of planar NWs and their integration into various functional devices^12,13,15^ that are being fabricated with significantly lower number of stages and preserves the NW pristine optoelectronic properties by saving the postgrowth alignment process. Nonetheless, the reported planar-grown NWs provide a limited optical response range, lacking the important near-infrared (NIR) range.

The demand for infrared (IR)-responsive semiconductors for IR photodetectors (PDs) has increased significantly during past decades due to their high significance in military and civilian technologies^22,23^ as night vision, advanced driver assist systems, travel security, and surveillance technologies. IR PDs are also useful in many other scientific analysis fields and methods, such as astronomy, spectroscopy, environmental monitoring, animal tracking, biological sensing, etc.^24^ There are three ranges of interest in the IR spectrum and several semiconductors commonly used as the active material in photodetectors in this range. The choice of the material is usually based on its optical response and band gap. Few examples are the III–V semiconductors, InGaAs^25^ and InAs,^26^ widely used for short-wave IR (SWIR) and midwave IR (MWIR). Specifically, the mid- and long-wave IRs (MWIR and LWIR), which are in the range of 3–5 and 8–12 μm, respectively, are at the center of interest for IR detection because they are in the atmospheric window, allowing the propagation of electromagnetic radiation such as IR radiation with minimal atmospheric absorption.^23^ These MWIR and LWIR photodetectors are based on II–VI semiconductors, notably mercury-cadmium-telluride (MCT or Hg_1–*x*_Cd_*x*_Te), whose band gap is controlled by varying the Hg content in the CdTe lattice, ranging from 2 μm in SWIR to ∼10.6 μm in LWIR.^27^ Since some of the materials can be alternatives for the same spectral regimes, one can choose the channel material considering the efficiency of the fabrication process, pricing, and functionality. Therefore, HgCdTe is often considered the ultimate channel material for devices operating in Fourier transform IR instruments (FTIR).^28^ However, both III–V and II–VI semiconductors are usually grown on costly complementary metal-oxide-semiconductor (CMOS)-incompatible substrates that are limited to small areas. In addition, their dark current is relatively high at high temperatures like room temperature, preventing efficient work under such conditions.^29^ Semiconductor NWs are expected to be advantageous candidates to address and overcome some of the mentioned challenges. NWs are strain and lattice mismatch tolerant owing to their low dimensions, relieving the strain elastically close to their surface.^30^ This allows versatility in the choice of cheaper and larger substrates. In addition, NWs have already shown a submicrosecond response to modulated light, with a high on–off ratio^31^ and ultrafast SWIR PDs,^32^ suggesting fast operation at room temperature.

CdTe is a semiconductor with optical response in the near-infrared (NIR) regime, attracting considerable research interest due to its high absorption coefficient and direct narrow band gap of ∼1.5 eV. Several methods yielded a vast repertoire of CdTe one-dimensional (1D) nanostructures^33−37^ integrated into functional PDs.^38−41^ Some PDs showed high responsivity and gain, but relatively long response times (100 ms).^40^ Moreover, most of the nanowire-based PDs were based on a single nanowire and were not scalable to large arrays of small pixels. CdTe can also be a starting material serving as the template for controlled synthesis of ternary MCT alloys by introducing Hg^2+^ cations to the CdTe nanocrystal after the morphology and size, as well as any chosen substrate, were determined.^42^ When a sufficient amount of Hg^2+^ cations replace Cd^2+^ cations, the band gap changes drastically from 1.5 eV (CdTe) to virtually zero (HgTe), while the framework of the anionic lattice is preserved.^43^ The combination of a single-step synthesis of CdTe NW arrays with the robustness of the cation exchange tool allows the efficient integration of photodetectors based on CdTe and Hg_1–*x*_Cd_*x*_Te with optimized performance and responsivity to any chosen range of the IR spectrum.

In this paper, we apply a Au-catalyzed, bismuth-assisted vapor–liquid–solid (VLS) growth of surface-guided CdTe NW arrays on two sapphire substrates: faceted annealed M (11̅00), and flat R (11̅02) substrates, exhibiting graphoepitaxial and epitaxial control over the NW orientation, respectively. The crystal structure and elemental composition were determined using transmission electron microscopy (TEM), energy dispersive X-ray spectroscopy (EDS), photoluminescence (PL), and Raman spectroscopy. The NWs exhibit growth with preferred orientations and a biphasic composition. To examine the photodetection performance of the NWs, they were parallelly integrated into an array of PDs by using a single photolithographic fabrication step. The devices were examined with a NIR-785 nm laser and exhibited high performance with an on/off ratio on the order of ∼10^4^ and response times of 90 and 104 μs for the rise and fall times, respectively. To the best of our knowledge, these are the shortest response times reported for PDs based on 1D CdTe nanostructures. Furthermore, CdTe NW arrays were converted to Hg_1–*x*_Cd_*x*_Te NW arrays by cation exchange, dipping the sample in a Hg^2+^ ion solution. After this cation exchange, the photoluminescence peak position shifted from 800 to 830 nm, corresponding to an estimated 2% exchange of Cd by Hg. This work demonstrates the potential integration of efficiently synthesized CdTe NWs into fast, room-temperature operational IR photodetectors with a tunable band gap.

## 2 Experimental Section

### 2.1 CdTe NWs Synthesis

The CdTe NWs were synthesized in a home-built chemical vapor deposition (CVD) system composed of a two-zone horizontal tube furnace. A 10:1 mixture of CdTe powder (99.99%, Sigma-Aldrich) and Bi powder (99.99%, American elements) was placed in the center of the hot first zone furnace. Commercial sapphire substrates (Roditi International Inc.) were used for the growth of CdTe NWs; *M*-plane sapphire was annealed for 24 h at 1600 °C in a single zone furnace under ambient pressure, and *R*-plane sapphire was used without further treatment. The wafers were patterned with 3 μm × 30 μm features of 5–8 Ȧ evaporated gold (Au, 99.999%, Holland Moran) using photolithography. The substrates were placed 33–40 cm downstream from the CdTe and Bi source mixture. The tube was initially purged under heating with three cycles of pumping to 6 mbar and filling with 450 sccm of N_2_ gas. Substrate temperatures range 400–430 °C and source temperatures range 580–620 °C. The synthesis was held for 1 h at a pressure of 10 mbar. The furnace was then allowed to cool to room temperature naturally.

### 2.2 Structural Characterization

The NW morphology, shape, size, and orientation were observed using a scanning electron microscope (Sigma 500 SEM, Zeiss). The analysis of the crystal structure and growth direction was done with a focused ion beam (FIB, FEI Helios 600 dual beam microscope, Thermo Fisher Scientific) to cut thin, electron-transparent lamellae in the NW cross section, which is sequentially observed with a high-resolution transmission electron microscope (HRTEM–Themis-Z). The TEM images were analyzed by extracting reduced fast Fourier transform (FFT) patterns from selected areas in the NW cross sections. The *d*-spacing values were compared to literature tables of bulk CdTe with an error < 5%. Elemental composition analysis was done using the same microscope with Super-X large solid angle X-ray energy dispersive X-ray spectroscopy (EDS) detector.

### 2.3 Optical Characterization

Photoluminescence (PL) and Raman measurements are done using a micro-Raman/micro-PL spectrometer (Horiba LabRAM HR Evolution). Two suitable laser lines were chosen for CdTe NW characterization: a 633 nm red laser (HeNe laser, Horiba) and a 785 nm laser (Diode laser). The lasers were focused on a single NW through a reflective objective lens ×100. Both emitted and scattered photons are collected by using the same objective and sent to a 600 or 1800 lines/mm grating.

### 2.4 Photodetector Fabrication and Characterization

A photolithography mask that is based on the predetermined catalyst features on the substrate was used to define electrodes with a 5 μm gap. Ti/Au electrodes with a ratio of 20/200 nm were deposited over the CdTe-guided NWs using an electron beam evaporator. The measurements were performed at room temperature and atmospheric pressure by using a 785-nm-high, single-frequency diode laser (Toptica photonics) as an illumination source. The measurements were conducted by using a Janis ST-500 probe system. Keithley 4200A system was used to apply source–drain DC bias and record *I*–*V* curves for different excitation powers. Keithley 4200-SCS system was used for pulsed measurements and fast recording of *I*–*t* curves using an optical chopper to shift the beam on and off the device.

### 2.5 Cation Exchange

A solution of 0.5 mmol of 3-mercaptopropionic acid (>99%, Sigma-Aldrich) in 50 mL of distilled H_2_O was prepared. The pH was raised to >10 using 0.5 M NaOH (>99%, Merck) solution. Another solution was prepared by dissolving 100 mg of Hg(ClO_4_)_2_·3H_2_O (>99%, Strem Chemicals Inc.) in 10 mL of H_2_O. The solutions were mixed, and the pH was fine-tuned to >10.5 using the same NaOH solution. The as-synthesized NWs on annealed *M*-plane sapphire were analyzed optically, as explained previously and then immersed for 24 h in the solution. The sample was then washed with H_2_O, and the PL/Raman spectra were collected from the same wires again.

## 3 Results and Discussion

**Figure 1:**
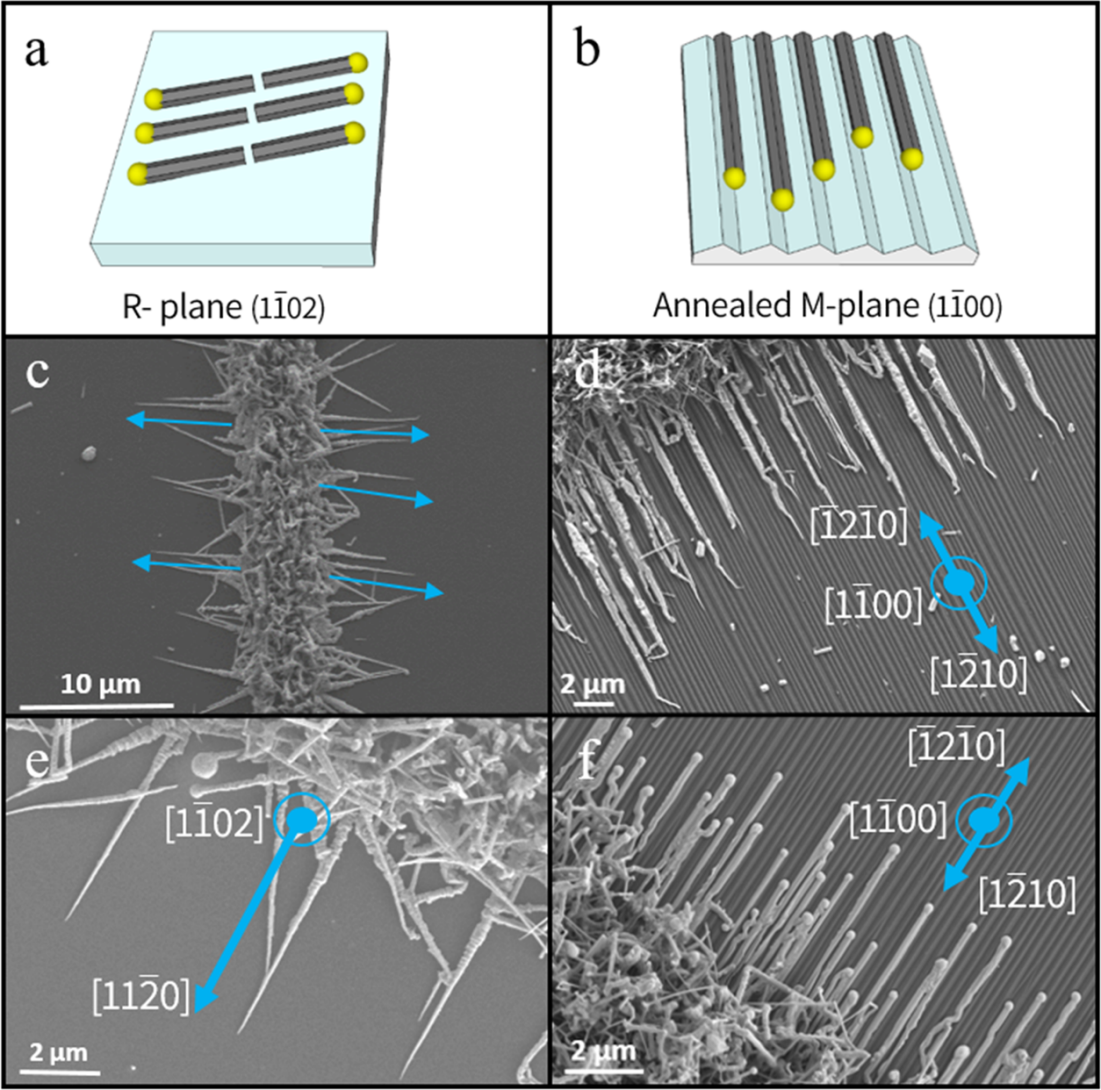
Guided growth of planar CdTe NWs. (a) Schematic of the growth of CdTe NWs on flat *R*-plane (11̅02) sapphire and (b) on faceted annealed *M*-plane (11̅00) sapphire. (c) Scanning electron microscope (SEM) image of guided CdTe NWs on *R*-plane sapphire and (d) on annealed *M*-plane sapphire. (e) Higher-magnification SEM image of guided CdTe NWs on annealed *R*-plane sapphire. (f) SEM image of CdTe NWs on annealed *M*-plane sapphire, grown with different morphologies. Here, the catalytic activity is well-observed at the tip of the NW. The blue arrows in all images show the growth orientation of the NW array with respect to the substrate used in the experiment.

The growth of the planar CdTe NWs was done in a chemical vapor deposition (CVD) system composed of a two-zone tube furnace. Gold (Au) patterns were created using a lithographic process and physical vapor deposition, supplying control over the NW’s position by preliminary positioning of the VLS catalyst. The patterned samples were held downstream to a 10:1 CdTe/Bi powder mixture with N_2_/H_2_ as the carrier gas at 10–15 mbar reduced pressure. Bi is a relatively known cocatalyst for the growth of II–VI semiconductor NWs. It decreases the melting point of the catalytic droplet, promoting its essential liquid state for VLS growth.^44,45^ This growth process yields aligned planar NWs in controlled positions and with specific, reproducible orientations, and the NWs are presented in Figure 1.

**Figure 2:**
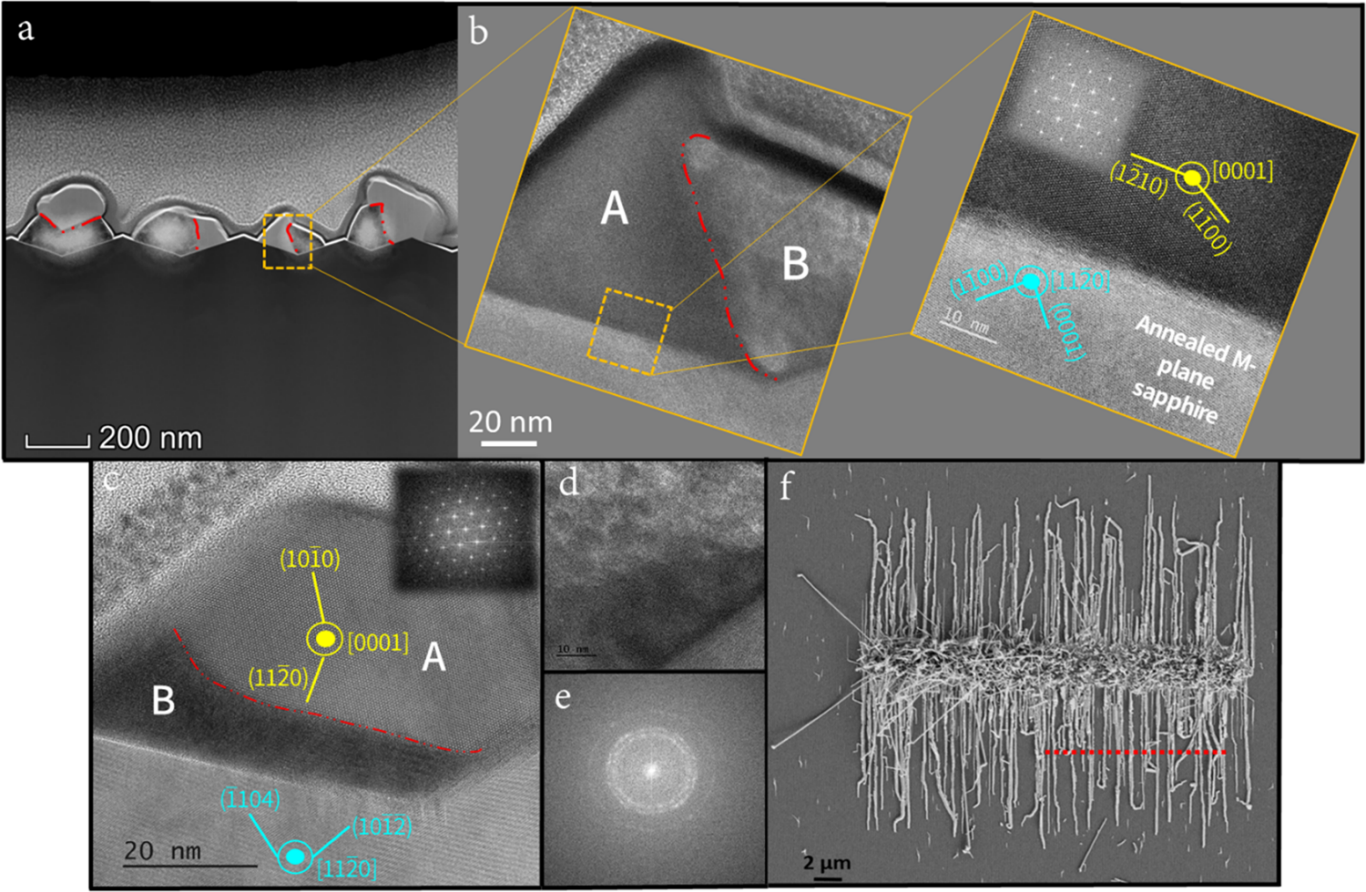
Crystal structure and crystallographic orientation of guided CdTe NWs on annealed *M*-plane sapphire. (a) TEM image across a thin, electron-transparent lamella made by cutting a set of NWs grown on annealed *M*-plane sapphire. The dashed red lines indicate the boundaries between the two distinct phases A and B. (b) Higher-magnification cross-sectional TEM images of a guided CdTe NW showing the A and B phases and the crystallographic orientation of phase A (yellow) with respect to the sapphire substrate (cyan). (c) TEM image of an additional NW from a different lamella showing similar phase separation and growth directions. Insets are the extracted FFT images taken from the crystalline phase. (d) High-magnification HRTEM image of the polycrystalline region of the NW presented in part (b), scale is 10 nm. (e) Extracted FFT image of the phase presented in part (d). (f) SEM image of a typical line profile guiding the lamella cut by FIB.

The planar CdTe NWs grow primarily at the edges of the patterned gold pads, as their approach to the surface drives their growth. The higher yield of nonplanar NWs covering the entire pad stems from the high density of dewetted gold nanoparticles in that region. These, as explained, are not the center of interest in the scope of this work and may be removed simply by sonicating the sample for a few seconds. This stage leaves behind the planar NWs and is followed by further characterization of the NWs and their integration into a NIR photodetector. The CdTe NWs typically grow with lengths of 3–12 μm and thicknesses of 50–300 nm. Most samples show uniform semicylindrical or polygonal prismatic shapes exhibiting the known hallmark of the VLS growth mechanism, which is the catalyst droplet at the tip of each NW (Figure 1f). Other NWs appeared to be tapered (Figure 1e). In this case, the catalytic droplet is either missing or too small to be seen. Tapered NWs are often observed when there is a dual growth mechanism. Alongside the elongation of the NW via the VLS mechanism, the already grown NW part gains thickness by adding precursor atoms via the vapor–solid (VS) mechanism.^46^ In order to characterize the crystal structure and crystallographic orientations of the NWs, a thin, electron-transparent lamella was prepared by using a focused ion beam (FIB) with sequential observation in a high-resolution transmission electron microscope (HRTEM) equipped with an accurate EDS detector. These data are also important for the investigation of the NW–substrate relations. The prepared lamellae for CdTe NWs on annealed *M*-plane sapphire can be seen in Figure 2a,b in three hierarchical magnifications.

### 3.1 Guided Growth of CdTe NWs on Annealed *M*-Plane (**101̅0**) Sapphire

The growth of CdTe NWs on annealed *M*-plane sapphire yields well-aligned NWs with a high yield of planar NWs with controlled orientations. Since *M*-plane (101̅0) sapphire is thermodynamically unstable,^47^ it undergoes spontaneous faceting when annealed in air at high temperatures (1600 °C). The more stable S (101̅1) and R (11̅02) facets exposed, creating V-shaped nanogrooves to the *A* direction, ±[12̅10], with characteristic depth, width, and pitch distance determined by the specific annealing temperature. In that case, the guided growth of the NWs is dictated by the graphoepitaxial effect, driven by minimizing the surface energy of the NW, enabling a surface-adjacent growth of the NWs as observed previously for a series of II–VI compounds.^11,13−16^ Cross sections of the NWs were observed in HRTEM and their crystal structure and growth direction were solved by extracting fast Fourier transform (FFT) images from certain regions following comparison with the characteristic *d* spacings of different plane families within the lattice. All comparisons were made with an error range of less than 5%. All observed NWs were found to grow in a biphasic structure, as depicted in Figure 2a and with higher magnification in Figure 2b. One phase is a single-crystal wurtzite (WZ) CdTe phase (denoted in Figure 2 as A), where all examined NWs grow along the *c*-axis [0001] (Figure 2b,c); the second phase (denoted as B in Figure 2) shows a characteristic diffraction of a polycrystalline phase (Figure 2d,e). Figure 2d shows an HRTEM image of phase B with no clear fringes of atomic planes. This phase’s character becomes clearer by extracting FFT from this selected area, yielding a characteristic pattern of a polycrystalline material as shown in Figure 2e.

**Figure 3:**
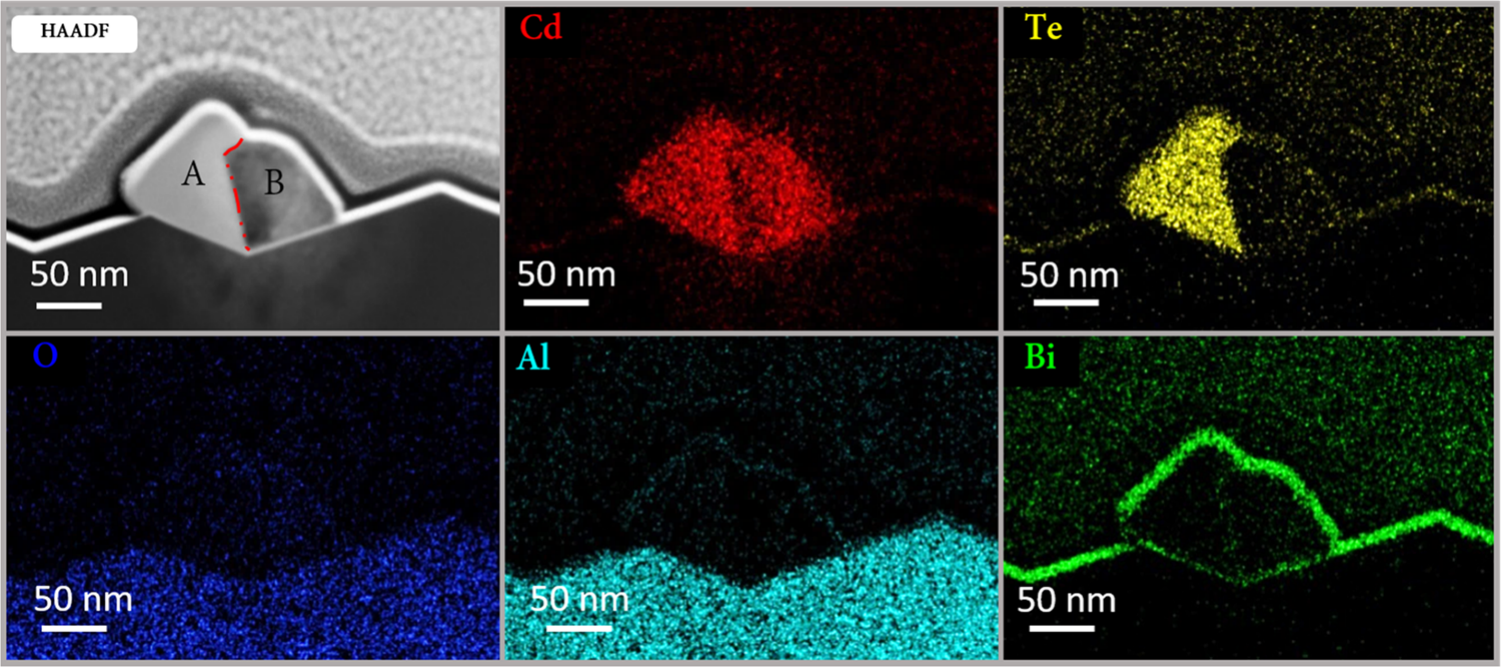
Elemental composition of CdTe NWs on annealed *M*-plane sapphire. High-angle annular dark field (HAADF) image of the wire and cadmium (red), tellurium (yellow), oxygen (blue), aluminum (cyan), and bismuth (green) maps are presented.

The chemical composition of the NWs was examined using EDS in order to understand their biphasic character and the chemical composition of each phase. Elemental maps are shown in Figure 3. The crystalline phase is composed of Cd and Te, and the polycrystalline phase shows high cadmium and oxygen contents. Whereas in the crystalline phase the Cd/Te ratio approaches ∼1:1 and is stoichiometric the polycrystalline phase is composed of cadmium and oxygen with a ratio of Cd/O that varied from one NW to another, and corresponded to a range of ratios 3:2–1:1, with a negligible amount of Te. This oxide with an intermediate stoichiometry may be attributed to the formation of CdO/Cd_2_O/Cd_3_O_2_ cadmium-based oxides caused by air leakage into the system. Nonstoichiometric deposition of CdTe structures from the gas phase is a well-known phenomenon, and the sensitivity of the final chemical composition to the synthesis conditions was already widely explored for thin films.^48,49^ In our case, the temperatures applied during the synthesis could induce a Te-deficient atmosphere, where the excess of Cd is compensated for by the small amount of O_2_ leaked into the system. Different diffusion and evaporation rates of the counterions can induce such a kind of atmosphere. For this reason, a Cd-rich polycrystalline phase with different oxidation states is observed. Bi turns out to accumulate in the catalytic droplet (see Figure S3), as expected from a cocatalyst, but also on the NWs’ or substrate’s surface, as can be seen in the Bi map in Figure 3, and its deposition is yet to be controlled.

### 3.2 Guided Growth of CdTe NWs on *R*-Plane **(11̅02)** Sapphire

**Figure 4:**
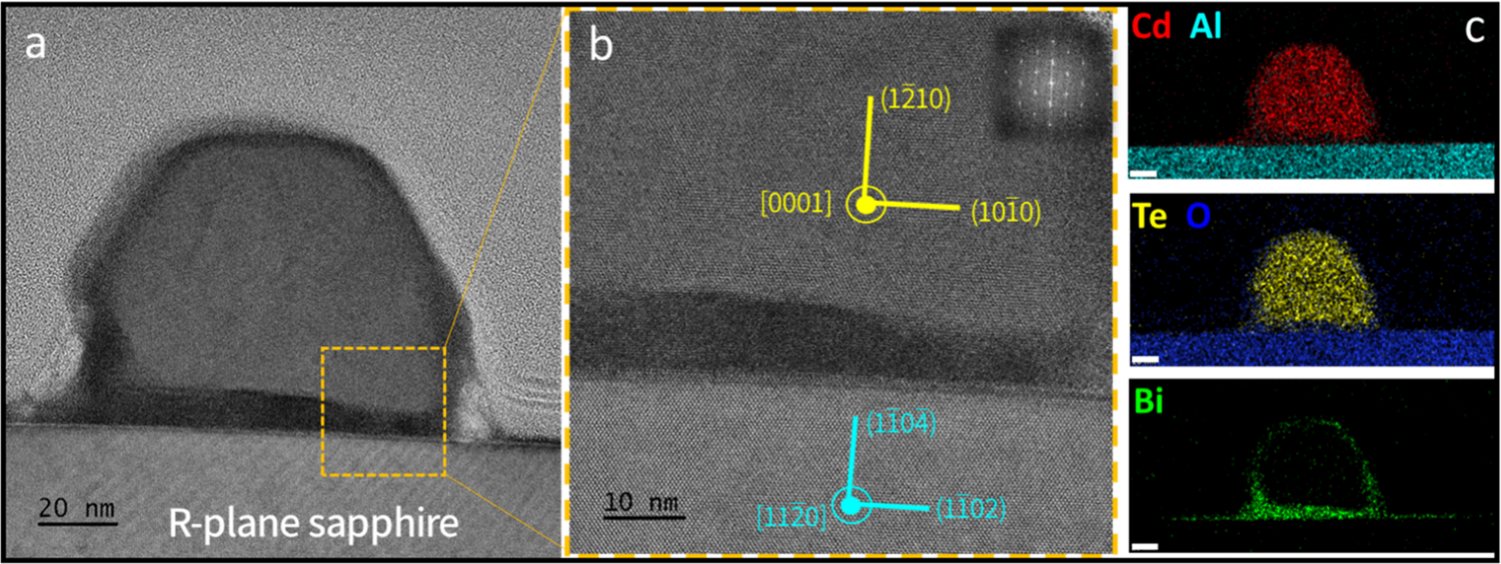
Crystal structure and orientation of guided CdTe NWs on *R*-plane sapphire. (a) TEM image of a typical NW cross section. (b) Higher-magnification TEM image with atomic resolution. Inset is the FFT pattern extracted from the NW and *R*-plane sapphire interface. (c) EDS elemental maps of a typical NW grown on *R*-plane sapphire. Cadmium (red), tellurium (yellow), oxygen (blue), aluminum (cyan), and bismuth (green) maps are presented. All scales are 20 μm.

The guided growth of CdTe NWs was also examined on atomically flat *R*-plane sapphire (11̅02). Here, we found several directions along which the NWs grow, each with a different yield. By focusing on a group of NWs that grow in the same crystallographic orientation, as depicted in Figure 1c, a thin lamella was cut and the NWs were explored using TEM and EDS. A typical NW cross-section TEM image is presented in Figure 4a. The observed NWs tend to grow with a WZ phase crystalline core of CdTe along the ±[112̅0] direction, with a polar growth axis [0001] (Figure 4b). This growth along the NW *c*-axis is not surprising and was already observed for other II–VI semiconductor NWs, such as ZnSe,^15^ CdSe,^13^ and ZnS,^11^ although these grow in other crystallographic orientations. This result strengthens our understanding that the substrate does not simply dictate the orientation of the NW, but it is also the growing material that plays an important role. Since the guided growth mode is epitaxy, the crystal’s growth is primarily driven by the minimization of the lattice mismatch between the growing material and the substrate. This principle is supported by calculating the transversal and longitudinal CdTe NW|| sapphire lattice mismatch. The transversal (12̅10)||(11̅04̅) mismatch stands for 0.1% and the longitudinal (0001)||(112̅0) mismatch of 0.6%. These small lattice mismatch values explain the preferred growth of CdTe with this specific orientation.

In addition to the crystalline core, another crystalline layer is seen with darker contrast in the TEM image in Figure 4b. According to the extracted FFT from the area defined by the second phase, we found that the phase is also crystalline as it shows a periodic FFT pattern. Nevertheless, the periodicity we have found in the particular measurement alignment is limited to a single direction, which was not enough for the determination of the structure and orientation of this phase (see Figure S2), and the crystallographic character of this layer is yet to be determined. We observed this layer on the circumference of most of the CdTe NWs. This seems general since it was also found in a nonplanar NW cross section (Figure S3). The EDS elemental maps, presented in Figure 4c, reveal that this phase is composed mainly of Bi, with small oxygen traces. Bi originates from the VLS catalytic droplet and Bi vapor atmosphere since it is a cocatalyst to gold. Regarding the crystalline core phase, the Cd/Te ratio is ∼1:1 with negligible oxygen content. The growth of planar CdTe NWs via two modes of guided growth extends the generality of the guided growth approach to an additional important II–IV semiconductor and creates a convenient platform for the integration of CdTe NWs into functional devices for IR technology.

### 3.3 Optical Properties of Guided CdTe NWs on Annealed *M*-Plane and on *R*-Plane Sapphire

**Figure 5:**
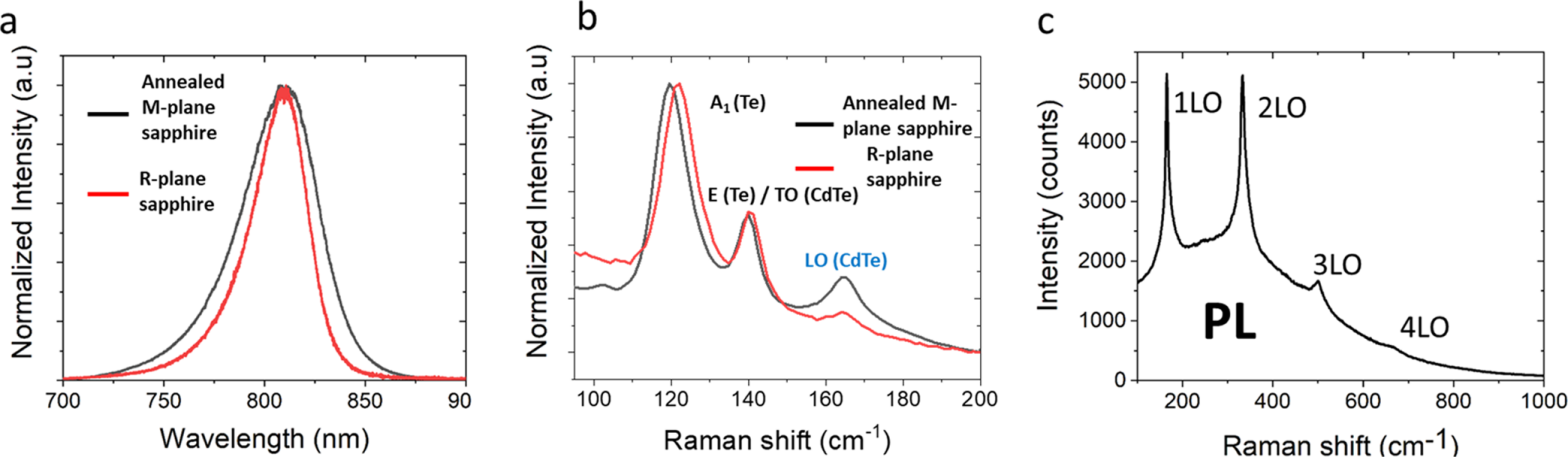
Optical properties of guided CdTe NWs. (a) Normalized photoluminescence (PL) spectrum of a single CdTe NW grown on annealed *M*-plane sapphire (black) and on *R*-plane sapphire (red) collected with a 633 nm laser excitation, both centered around 810 nm. (b) Normalized Raman spectrum of a single CdTe NW grown on annealed *M*-plane sapphire (black) and on *R*-plane sapphire (red) collected using a 633 nm excitation exhibiting the well-known A_1_ and E modes of hexagonal Te at ∼120 and 140 cm^–1^, respectively, and the exclusive characteristic LO phonon mode of CdTe at ∼165 cm^–1^. (c) Close-to-resonance multi-LO phonon Raman spectrum of single NW grown on annealed *M*-plane sapphire collected using 785 nm excitation. We observed the multi-LO phonon cascade up to the fourth order due to the electron–phonon interaction.

The optical properties of the CdTe NWs are key for their integration into efficient optoelectronic devices, especially photodetectors, where the physical processes generated by the electromagnetic radiation determine the device’s performance. The optical characterization of the NWs was done in order to have a clear sight at the near band-edge (NBE) emission and thus on the spectral regime of the CdTe optical response. Structural information and an additional understanding of the optical properties are achieved by looking at the inelastically scattered photons. Photoluminescence (PL) and Raman measurements were done using a micro-Raman/micro-PL spectrometer (Horiba LabRAM HR Evolution). Two suitable laser lines were chosen for CdTe NW characterization near the expected band gap of ∼1.5 eV: a 633 nm (1.96 eV) red laser (HeNe laser, Horiba) for PL and Raman spectroscopy and a 785 nm NIR (1.58 eV) diode laser for resonant Raman spectroscopy. The PL spectrum was collected from a single CdTe NW grown on annealed M-plane sapphire and on *R*-plane sapphire and is shown in Figure 5a. The PL spectrum from NWs grown on annealed *M*-plane sapphire exhibits relatively broad peaks differing from one NW to another, as seen in Figure S4. The spectra are centered at a range of wavelengths between 800 and 826 nm, typically around the corresponding energy gap of 1.53 eV, which is comparable with the bulk CdTe band gap. No quantum confinement effects are expected since the CdTe exciton Bohr radius is ∼7 nm,^50^ orders of magnitude smaller than the NW average radii. These fluctuations can thus be attributed to small variations in the chemical composition of the NWs, as discussed before. The shifts in the PL spectrum could also stem from strain effects in the NWs, as previously observed for ZnS NWs.^11^ A fit to a Voigt line shape (a combination of Lorenz and Gauss fittings) was done to estimate the width of the spectrum, yielding two width parameters, the Gauss width, *W*_G_, and the Lorenz width, *W*_L_. For the NWs on annealed *M*-plane sapphire, typical widths of *W*_G_ = 32.3 ± 0.4 nm and *W*_L_ = 22.2 ± 0.5 nm were concluded, while for the NWs grown on the R-plane, the values were lower with *W*_G_ = 22.2 ± 0.4 nm and *W*_L_ = 17.5 ± 0.5 nm. The fitting curves are presented in Figure S6. It is also directly observed in Figure 5a that the spectrum of the NWs grown on *R*-plane sapphire is narrower and is directly in line with our EDS analysis, which supports higher crystal purity for the NWs grown on *R*-plane sapphire, lacking the oxidized cadmium phase in the NW. Different spectra collected from several NWs grown on *R*-plane sapphire were also narrowly distributed around 810 nm, as can be seen in Figure S4.

The Raman spectrum collected from a single CdTe NW grown on an annealed *M*-plane and on *R*-plane sapphire using a 633 nm laser further supports the crystal structure and composition, as shown in Figure 5b. The peaks in ∼120 and ∼140 cm^–1^ are related to the A_1_ and E modes of hexagonal Te, respectively, arising from Te clusters on the surface, stem from laser heating and dangling bonds that induce Te–Te bonds in Te clusters on the surface.^51^ The peak at 140 cm^–1^ is a superposition of the E (Te) mentioned above, with the transversal optic (TO) mode of CdTe. The fingerprint, exclusively related to CdTe lattice phonons, is the peak at ∼165 cm^–1^, assigned as the longitudinal optic (LO) mode of CdTe.^52^ Notably, the peak position of all three Raman modes, characteristic of vapor-phase-grown CdTe, i.e., the A_1_ and E modes of Te and the LO mode of CdTe, remains consistent and independent of the substrate choice. However, a slight disparity in the intensity of the peaks, mostly apparent in the LO mode at 165 cm^–1^, can be attributed to the interaction of different CdTe facets with the incident light.^51^ This variance may potentially arise from the compositional differences, which vary from one NW to the other and were pointed out before in our TEM and EDS analysis. Another Raman spectrum was taken under close-to-resonance conditions using a 785 nm laser. The spectrum is presented in Figure 5c. In this case, electron–phonon interactions induce a series of intensified peaks shifted by sequential ω_LO_ gaps between them. These multiphoton resonant peaks at 165, 330, 495, and 660 cm^–1^ are respectively assigned as first, second, third, and fourth orders of the LO mode in CdTe. The exciton relaxation mediated by several orders of LO phonons was observed before for many II–VI direct band gap semiconductors such as CdS,^53^ ZnO,^54^ and CdTe^55^ and was related to a good optical quality of the material.^54^ These results, with the band-edge emission of the NWs centered at ∼1.53 eV in the NIR regime, in addition to clear Raman signals, are a strong indication that even though the NWs contain a polycrystalline phase, they have an optically responsive crystalline phase of CdTe that can be integrated into high-performance optoelectronic devices.

### 3.4 Optoelectronic Characterization of CdTe NWs on Annealed *M*-Plane Sapphire

**Figure 6:**
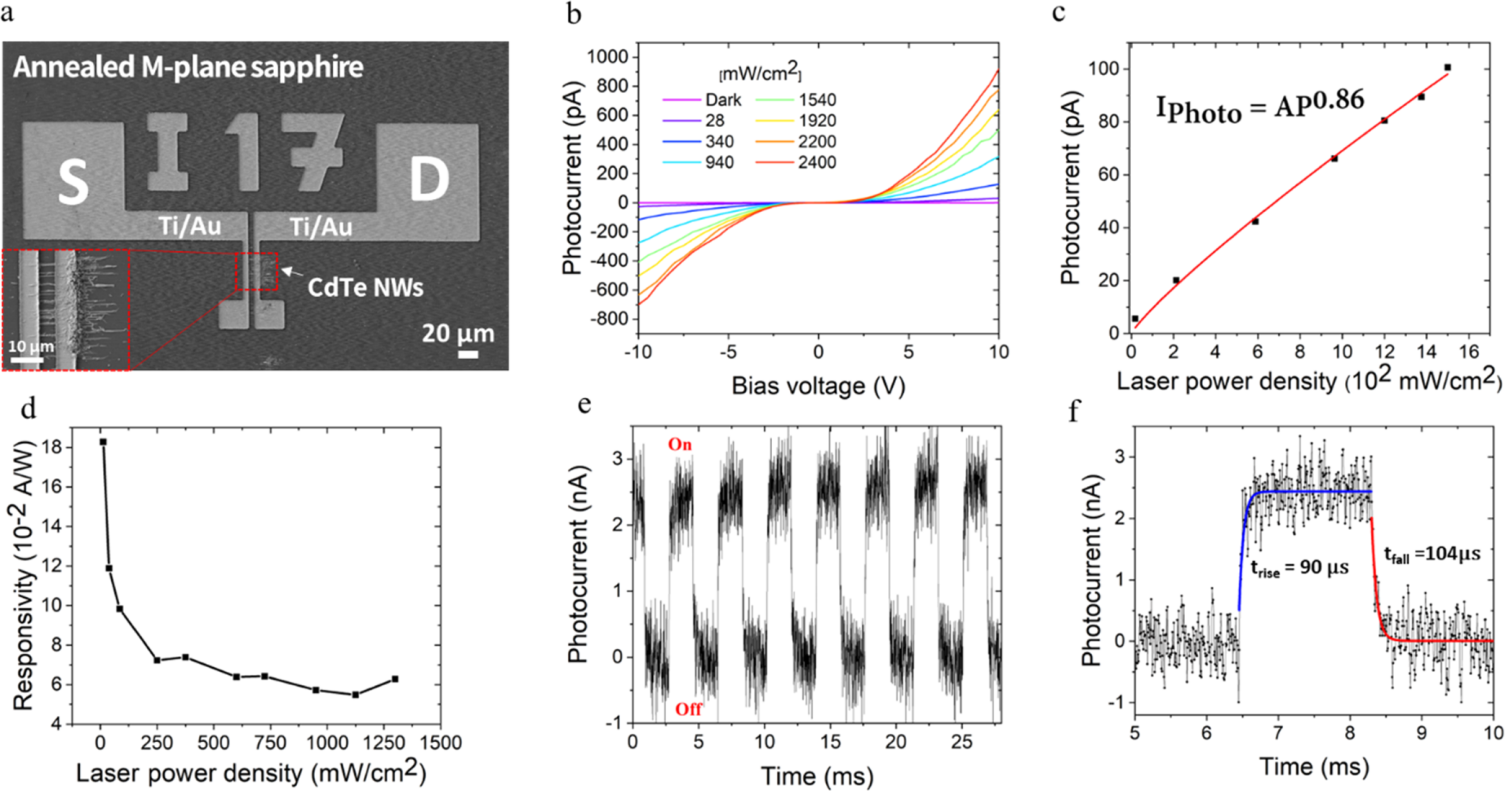
Optoelectronic characterization of guided CdTe NW arrays. (a) SEM image of a characteristic device with 5 μm gap source/drain Ti/Au electrodes connected by ∼12 NWs. (b) *I*–*V* curves measured under sweeping voltage of −10 to 10 V of several 785 nm laser power densities in the range of 0–1500 mW cm^–2^. (c) Common power fit of the photocurrent as a function of laser power density, yielding the function *I*_photo_ = *AP*^0.86^. (d) Responsivity changes with increasing laser power density. (e) Photoresponse of the device under light modulation for eight on–off cycles (270 Hz, 30 V bias, and 475 mW cm^–2^). (f) One cycle of on–off switching is presented in panel (e) with higher magnification. The single exponential fittings of the rise and fall regimes are presented in blue and red, respectively.

The potential of guided CdTe NW arrays as the active component in optoelectronic devices was examined by fabricating two terminal photodetector arrays based on NWs grown on annealed M-plane sapphire. Each device consists of 5–12 NWs bridging two Ti/Au (20/200 nm) source–drain electrodes with a 5 μm gap. A typical device is presented in Figure 6a, and an array of devices, as well as a higher-magnification SEM image of a device channel, are presented in Figure S7. The photoresponse to IR illumination was examined using a 785 nm diode laser, illuminating the device with a series of laser power densities (including dark) with bias voltage sweeps in the range of −10 to 10 V. *I*–*V* curves describing the photocurrent with increasing light densities are shown in Figure 6b. The expected current increase due to increasing laser power densities reveals shallow dark currents (less than 2 pA at 20 V bias). This is also manifested by the high ratio between the photocurrent and dark current, which can also be termed as the on/off ratio of the photodetector ranging from 6.43 × 10^2^ to 1.38 × 10^4^, at 10 V bias and 1500 mW cm^–2^ illumination. Differences from one device to another are again related to the differences in NW composition and the different numbers of NWs in each device. The behavior of the photocurrent and its increase with increasing light densities is often described using a simple power law, *I*_photo_ = *AP*^θ^. This commonly used function yields a preexponential coefficient, *A*, that depends on the illumination wavelength; and the power, θ, which is strongly related to the charge carrier lifetime. The fit to our results is presented in Figure 6c, yielding θ = 0.86 for the CdTe NWs. θ values ranging from 0.5 to 1.0 indicate bound states distributed in such a way that their concentration decreases farther from the band edges. This behavior was explained successfully by Rose,^56^ who stated that the lifetime of the free charge carriers decreases with increasing light intensity. The thermal equilibrium is interrupted upon excitation, and two quasi-Fermi levels for electrons and holes are created. When the semiconductor is illuminated with higher laser power density, the quasi-Fermi levels are pulled toward their respective band edges, leaving behind additional ground states, which serve as recombination centers at the expense of shallow traps. This increase in recombination center concentration shortens the carrier lifetime, as manifested in the sublinear proportionality. Another two standard parameters describing the device performance are the responsivity (*R*_λ_) and gain factor (*G*). Responsivity represents the current generated per light density unit and effective illuminated device area and is calculated as
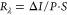
1where Δ*I* is the net current induced by light illumination (*I*_photo_ – *I*_dark)_, *P* is the laser power density, and *S* is the effective area of the illuminated device. In our case, *S* is estimated by the product of electrode gap × NW diameter × number of NWs. The gain factor is among various parameters describing the number of generated electrons participating in the conduction with respect to the number of hitting photons. It is calculated according to
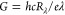
2where *h* is Planck’s constant, *c* is the speed of light, *e* is the electron charge, and λ is the illuminated light wavelength. In our measurements over three devices, those that yielded detectable dark current, the responsivity ranged at (48–77) × 10^–2^ A W^1–^, and the proportional gain ranged from 0.76 to 1.22. These values are comparable to those of ZnS^57^ and ZnSe^58^ nanobelts, as well as CdTe quantum dots (QDs).^33^ We relate these values to a relatively high concentration of deep traps arising from oxygen impurities in the CdTe crystal, as mentioned before. These traps increase the NW resistivity and lower the generated steady-state photocurrent. The proposed model for the charge carrier lifetime shortening can also explain the responsivity decay with increasing laser power density, as shown in Figure 6d.

The response time of the device is an additional essential figure of merit regarding the device performance in both fundamental and applicative essence. Different applications frequently demand a fast device response, and the response time is a key concept in understanding the charge carrier dynamics in the channel material. We investigated the device photoresponse to modulated IR illumination. The measurement was done using an optical chopper that shifts the laser beam on and off the device with a frequency of 270 Hz, a laser power density of 475 mW cm^–2^, and a bias voltage of 30 V. Figure 6e presents eight on–off cycles, where the current periodically switched due to the excitation and decay of charge carriers in the device channel. One cycle is magnified and closely analyzed, as presented in Figure 6f. The calculations of the rise and fall time, which are defined as the time needed for the device to rise from 10 to 90% of the current maximal value and vice versa,^59^ respectively, were done using the exponential fitted function. The exponential fit is also presented in Figure 6f and the results for the two devices reveal a rise time range of 90–230 μs and a fall time range of 104–179 μs. The exhibition of a single exponential decay indicates the dominant mechanism controlling the carriers’ dynamics is their fast recombination and unstrapping. To the best of our knowledge, these are the shortest response times ever reported for CdTe nanostructure-based PDs. A comparison to other CdTe nanostructure-based PDs is presented in Table 1.

**Table 1:** Comparison of Device Performance of CdTe NWs’ Arrays with Other Reported CdTe-Based PDs

active nanostructure	λ (nm)	*P* (mW cm^–2^)	bias (V)	*I*_dark_	*I*_photo_	*t*_rise_	*t*_fall_	*R*_λ_ (A/W)	*G*	refs
CdTe single NW	400	0.55	10	6 pA	25 pA	0.7 s	1 s	80.1	2.5 × 10^4^	(38)
CdTe single μW	365	0.86	20	∼ 1 pA	60 pA	7.7 s	60 ms	n.a.	n.a.	(41)
CdTe nanoribbons	400	0.637	10	0.4 nA	1 nA	*1.1 s	*3.3 s	7.8 × 10^2^	2.4 × 10^5^	(33)
							17.1 s			
CdTe single μW	365	15**	20	0.3 nA	3 nA	6.2 s	13.3 s	n.a.	n.a.	(37)
CdTe single NW	633	4	1	0.01 pA	230 pA	<100 ms	<100 ms	6.9	n.a.	(40)
CdTe single NW	800	0.1	5	500 pA	2.1 nA	*6.12 s	*7.53 s	3.6 × 10^2^	5.56 × 10^2^	(60)
CdTe single NWs	white light (AM 1.5)	100	1	∼3 nA	940 nA	9.45 ms	9.98 ms	n.a.	n.a.	(39)
CdTe NWs array	785	1500	10	∼0.6 pA (noise level)	920 pA	90 μs	104 μs	0.5	0.9	this work

There are several possible reasons to explain the fast response of our devices, including the small size of the NWs, high bias voltage, and small transfer gap between the source–drain electrodes (5 μm).^31^ According to Rose,^56^ one of the bottlenecks is that the charge carrier dynamics in a photodetector are shallow trap states found close to the band edges. Charge carriers are expected to fill these traps during the rise induced by illumination and evacuate them when the light is switched off, causing the current fall. We found it reasonable that in our case, since the NWs are relatively big, surface states concentration is relatively low and does not play a significant role in the charge carrier dynamics. This, in turn, brings the observed fast response. We can also suggest that a limited role is related to the graphoepitaxial in-plane growth, which leads to a partial passivation effect of the NW surface area, as some facets are adjacent to the sapphire surface. To that we can also add a passivation effect of the surface states by the Bi thin films deposited on the NWs as shown in Figure 3. A similar passivation effect was previously shown in a paper by Naffeti et al. for Si NWs, decorated by Bi nanocrystals.^61^ This study demonstrated that the deposition of Bi nanocrystals on top of Si NWs induces a synergetic effect, augmenting the optical properties of the NWs. The Bi coverage serves a dual purpose, acting both as a surface passivation agent and as an effective antireflector coating. By carefully controlling its thickness, a light-trapping phenomenon is achieved between neighboring NWs, resulting in an enhancement of the NWs’ photoluminescence and, possibly, an improved optical response.

Moreover, the surface passivation of the NW surface by Bi leads to a notable reduction in the surface-state concentration, further contributing to the enhancement of the optical performance. This dual functionality of the Bi thin layer showcases its role in optimizing the NW properties and underscores its potential for use in efficient optoelectronic devices. As a result of all mentioned factors, the main inhibitors for the PD fast response are eliminated and lead to the observed short rise and fall times, further demonstrating the advantages of the guided growth approach. From a bird-eye view, the presented case is not rare and exhibits a photodetector with a clear responsivity and response time trade-off,^62^ as we observed relatively low responsivity and gain but very short response times. This behavior is a direct result of different defect types in the NWs as explained by rose [ref (56)]. While having a relatively high concentration of deep traps that lowers the steady-state current and thus the responsivity and gain, the large size of the NWs induces lower significance of the surface state and sets the conditions for the photodetectors’ fast response.

### 3.5 Cation Exchange and Band Gap Narrowing in CdTe NWs on Annealed *M*-Plane Sapphire

**Figure 7:**
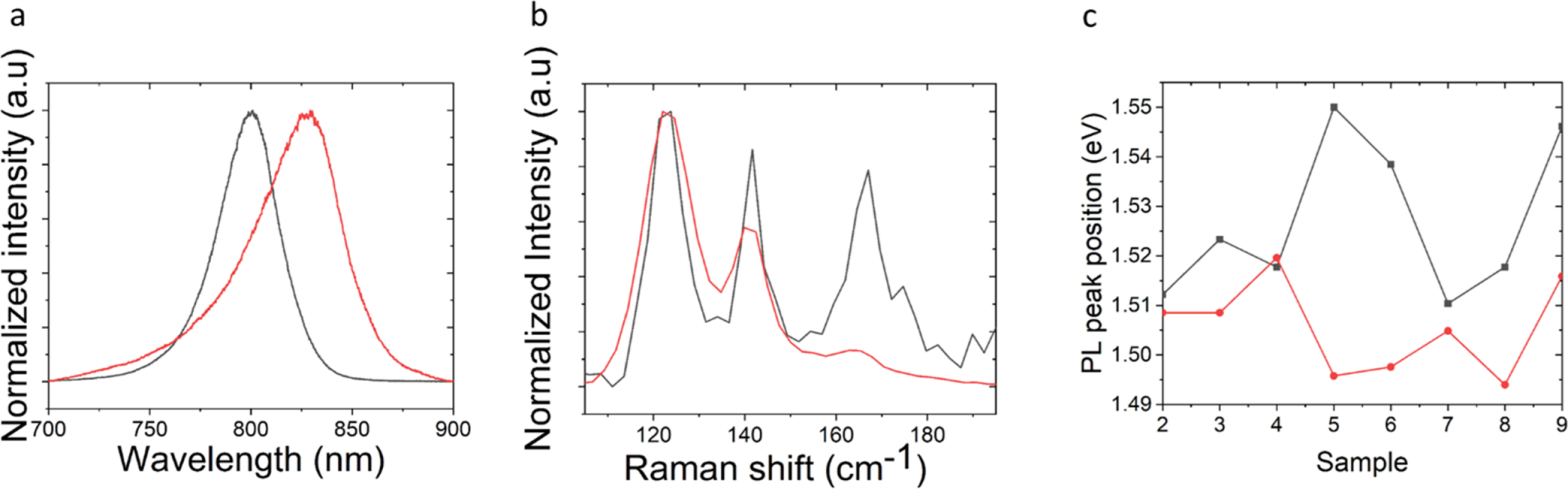
Cation exchange experiments on CdTe NWs grown in annealed *M*-plane sapphire. (a) PL spectrum of as-synthesized CdTe NW centered at around ∼800 nm (black) and red-shifted PL spectrum of the same NW after immersion of the sample in Hg^2+^ solution centered at around ∼830 nm (red). (b) Raman spectrum of as-synthesized CdTe NW with clear LO phonon peak at ∼165 cm^–1^ (black) and Raman spectrum of the same NW after immersion of the sample in Hg^2+^ solution (red) with a dramatic decrease in the LO phonon peak intensity. (c) Summary of NBE emission shifts of 8 NWs of CdTe immersed in Hg^2+^ solution. Red shift is observed with differentiation from one NW to another in the vast majority of the analyzed NWs.

In order to fulfill the potential of the guided CdTe NWs as a channel material in PDs for a wide IR range, mercury (Hg^2+^) cation exchange experiments were performed to narrow the band gap, extending the NWs’ optical response range to deeper IR regimes. Alloying of CdTe with HgTe was done before using several dissolved mercury compounds, interfacing various CdTe nanostructures including nanocrystals,^63^ QDs,^64^ and nanoplatelets.^65^ Those attempts yielded different narrow band gap ternary alloys with the general formula Hg_1–*x*_Cd_*x*_Te. As far as we know, there have been no attempts for cation exchange on planar CdTe NWs. This robust tool suggests an opportunity for controlled wavelength modulation, potentially covering the entire IR spectral regime. Planar CdTe NWs were grown on annealed *M*-plane sapphire, similar to previous samples. The ∼0.5 cm × 0.5 cm sample containing several arrays of CdTe NWs was characterized using the micro-PL/Raman system. Ten NWs were chosen from different locations across the wafer, and their optical properties were documented. The sample was immersed then, in an aqueous solution of 3.67 mM Hg(ClO_4_)_2_·3H_2_O with 8.33 mM 3-mercaptopropionic acid serving as a ligand with pH > 10. The ligand mediates the Hg^2+^ insertion into the crystal and pulls out Cd^2+^ ions, further promoting the cation exchange as previously proposed for CdTe quantum dots.^64^ After 24 h, the sample was washed with distilled water and PL and Raman spectra were taken from the same NWs documented before. PL spectra are an effective way to track the changes in the NBE emission of the resulting structure. A typical PL spectrum (normalized) of pristine single CdTe NW before (black) and after (red) exposure to mercury ions solution is presented in Figure 7a. A clear red shift together with line shape broadening can be seen. We attribute the broadening to defects created in the crystal during the cation exchange process. The adaptation of the ongoing growth of the Hg_1–*x*_Cd_*x*_Te crystal, which naturally prefers cubic phase, to the existing WZ phase of CdTe NWs, may induce stacking faults. In addition, uncontrolled diffusion processes of the ions repertoire in the solution may induce additional defects causing the exhibited line shape broadening, and particularly Hg ions that are distributed inhomogeniously into the NW’s crystal. Typical Raman spectrum (normalized) of pristine CdTe NW and the same NW after exposure to a mercury ion solution appears in Figure 7b. The characteristic LO peak of CdTe at ∼165 cm^–1^ loses its intensity dramatically after exposure of the NWs to the mercury solution. At the same time, the Te-related A_1_ and E modes remain approximately unchanged. This decrease in intensity indicates a significant structural change in the NWs. Figure 7c summarizes the PL results for eight undamaged NWs, showing band gap narrowing up to 55 meV. Figure S8 summarizes the energy dispersive spectroscopy (EDS) measurement collected from CdTe NWs after the cation exchange process in the Hg^2+^ ion solution. These results support the mercury insertion and distribution along the wires, as reflected in the mercury map in Figure S8-d. We treat the quantitative results ambiguously for the reason that we cannot say for sure what part of the signal comes from the wires and what part comes from Hg(ClO_4_)_2(s)_ and its byproduct sediments on the surface. A rough theoretical calculation of the molar fraction of Hg in the crystal is possible using the experimental equation suggested by Casselman et al. in 1982^66^ regarding the changes in Hg_1–*x*_Cd_*x*_Te band gap as a function of the Cd content, *x*, and temperature, *T*. They suggested the band gap obeys the following function
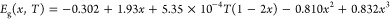
3

Since we assume small amounts of mercury, a calculation of the derivative around *x* = 1 (where [∂*E*_g_/∂*x*]_*x*=1_) at room temperature supplies a good estimation of the change in *x*. These calculations determine that the new NWs are composed of up to ∼2% (molar) of mercury. The main limitation for the exchange has to do with thermodynamics: while the CdTe NWs crystallize in the WZ phase, HgTe usually stabilizes in the cubic ZB phase. This thermodynamic limitation was also suggested to be the primary limit for the exchange of CdSe quantum dots into Hg_1–*x*_Cd_*x*_Se alloys.^67^ Another possible explanation for the partial cation exchange was suggested before for the system of CdSe–HgSe nanoplatelets by Ithurria et al.^68^ Since mercury ions are larger than cadmium, the Hg-insertion kinetics are inhibited and depend on the Cd vacancies that exist or are being created on the surface. Similar results to those presented for Hg (ClO_4_)_2_ were achieved using HgCl_2(EtOH)_ solution, and the main results of this part are shown in Figure S9. These promising results provide proof of concept of the cation exchange method on bare CdTe surface-attached NWs synthesized by CVD. As pointed before, the growth of MCT nanostructures via cation exchange is a robust tool allowed in the past, the compositional engineering of CdTe to a series of different MCT nanostructures displaying different Hg-to-Cd ratios, manifested in different optical properties.^64^ In our case, misfitting of the hexagonal CdTe phase to the preferred cubic phase of HgTe is suggested to limit the process. The future research will need to focus on both CdTe NW synthesis and cation exchange conditions in order to increase Hg concentration in the NWs. We believe that tuning the exchange experiment conditions, as well as controlling the CdTe NW phase, may extend the degrees of freedom related to MCT compositions and NWs suggested by our approach, corresponding with thermodynamic or kinetic considerations in the process. Overall, the two complementary, simple synthesis methods, as used and exhibited in this paper, pave the way for fast integration of narrow band gap NWs into IR active devices and, specifically, fast PDs based on tunable band gap planar NWs.

## 4 Conclusions

In summary, this work demonstrates the surface-guided growth of CdTe NW arrays with controlled orientations and growth directions on both annealed *M*-plane via graphoepitaxial guidance and *R*-plane sapphire via epitaxial guidance. The growth of CdTe NWs further generalizes the guided growth phenomenon and adds an important IR-responsive semiconductor to the growing list of guided semiconductor NWs. The NWs grow with a crystalline WZ CdTe core phase adjacent to another Cd-rich (in the case of the annealed *M*-plane) or Bi-rich (in the case of the *R*-plane) phase. The NWs exhibit clear optical properties, including the multi-LO phonon Raman scattering related to high optical quality with optical signatures associated with CdTe. The guided growth of the NWs allowed gain of control over their position, growth direction, and orientation, facilitating their integration into planar functional arrays of NIR PDs. The PDs exhibited extremely low dark current and, to the best of our knowledge, the shortest response times among other reported CdTe nanostructure-based PDs. These results further demonstrate the potential of guided NWs of IR-responsive materials as components in many different high-performance devices that are easily fabricated and have strong potential for scale-up. To extend the range of IR detection, the CdTe NWs were used as a template for creating Hg_1–*x*_Cd_*x*_Te using cation exchange with different mercury compounds. We observed a significant red shift in the NW band-edge emission of up to 29 nm, corresponding to a ∼55 meV band gap narrowing. These preliminary results show that the guided CdTe NWs are promising building blocks for high-performance PDs, holding the ability to further stretch their responsivity regime to longer wavelengths in the IR spectrum. This can answer the need for fast-response IR-responsive materials for efficient and easily fabricated narrow band gap devices.

## Supporting Information Available

Different guided NWs grown with the same crystallographic orientation (Figure S1); second phase of NWs grown on *R*-plane sapphire (Figure S2); nonplanar CdTe NWs grown with the zinc-blende phase (Figure S3); additional EDS maps of different CdTe NWs showing the generality of composition (Figure S4); distribution of PL emission of different CdTe-guided NWs (Figure S5); line shape fitting of the PL spectra of guided NWs (Figure S6); array of photodetectors fabricated on top of arrays of guided CdTe NWs (Figure S7); post cation exchange analysis of guided MCT NWs (Figure S8), and results of cation exchange with different mercury sources (Figure S9) (PDF)

## Abbreviations

MCT: mercury-cadmium-telluride
NWs: nanowires
VLS: vapor–liquid–solid
PD: photodetector
NIR: near-infrared
IR: infrared
SWIR: short-wave infrared
MWIR: midwave infrared
LWIR: long-wave infrared
HRTEM: high-resolution transmission electron microscope
EDS: electron dispersive spectroscopy
PL: photoluminescence
CVD: chemical vapor deposition
FFT: fast Fourier transform
WZ: wurtzite
NBE: near band-edge emission
TO: transversal optics
LO: longitudinal optics
