# Supplementary material for: Guided CdTe Nanowires Integrated into Fast Near-Infrared Photodetectors
Source: ACS Appl Mater Interfaces. 2024 Jan 4;16(2):2637–48. doi: 10.1021/acsami.3c15797 (PMC10797596; doi:10.1021/acsami.3c15797)
Supplement: Supplementary file 1 — am3c15797_si_001.pdf [file am3c15797_si_001.pdf]

Supporting Information

Guided CdTe Nanowires Integrated into Fast Near-Infrared Photodetectors

*Yarden Danieli[1], Ella Sanders[1], Olga Brontvein[2] and Ernesto Joselevich[1]**

[1] Department of Molecular Chemistry and Materials Science, Weizmann Institute of Science, Rehovot 76100, Israel

[2] Department of Chemical Research Support, Weizmann Institute of Science, Rehovot 76100, Israel

*Email: ernesto.joselevich@weizmann.ac.il

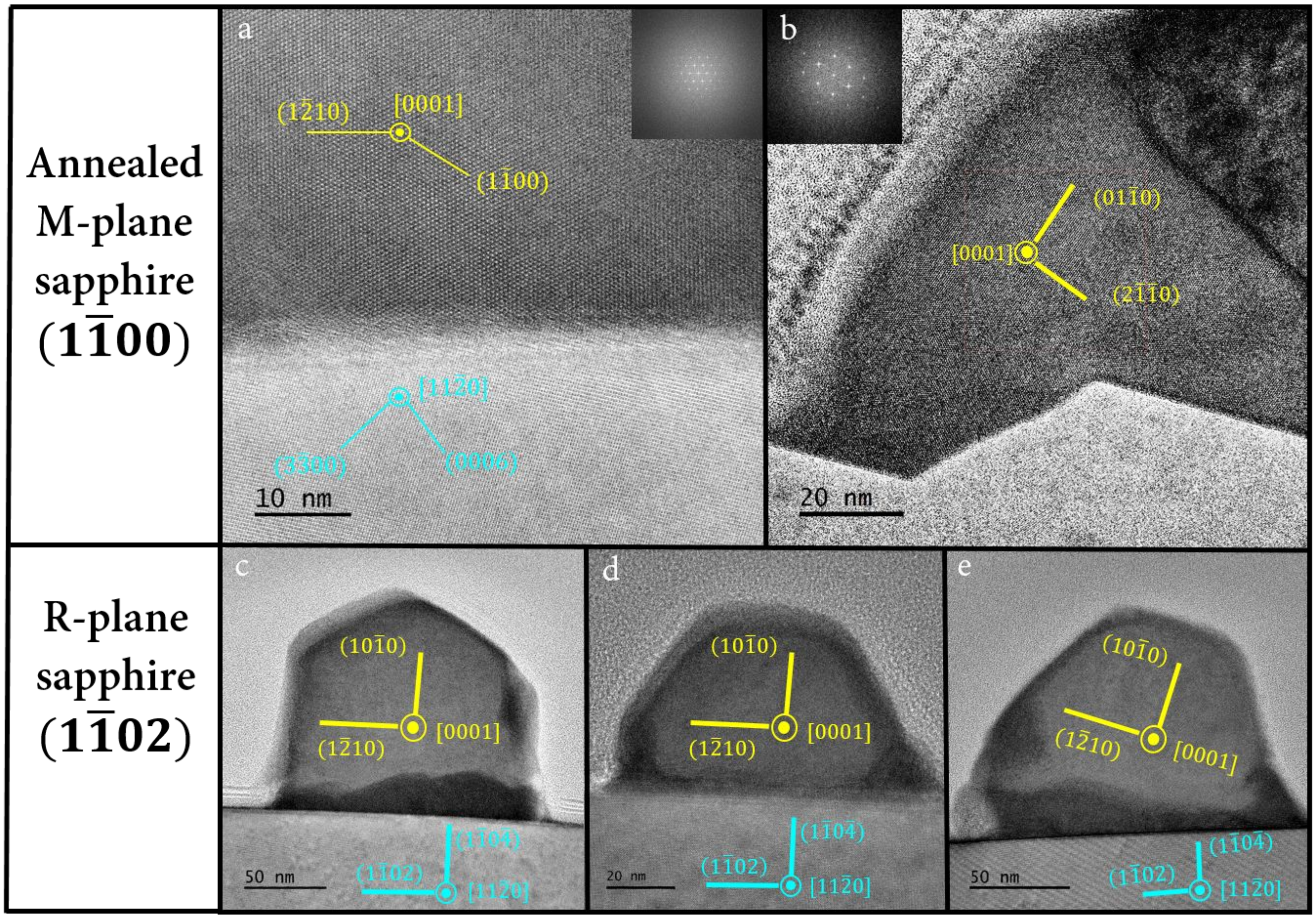

Figure S1. (a) and (b) Show Additional cross-sectional TEM images of CdTe NWs on annealed M-plane $(1\bar{1}00)$ and (c), (d), (e) on R-plane $(1\bar{1}02)$ sapphire. This analysis statistically supports the reported orientations for each plane, respectively. All the wires show the same growth direction. In the case of the epitaxial guided growth (on R-plane sapphire), all of the examined NWs exhibit uniform epitaxial relations to sapphire, with the same longitudinal and transversal planes.

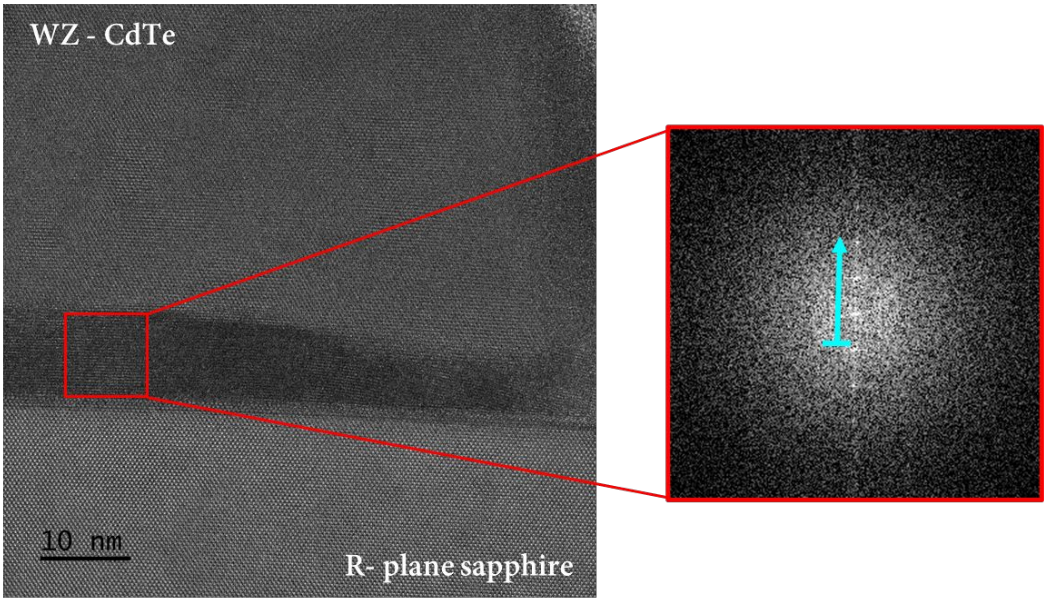

Figure S2. A TEM image of a WZ-CdTe grown on top of R-plane sapphire. the extracted FFT pattern, from the area defined by the red square shows periodicity along a single direction, which is not enough for the determination of the crystal structure and orientation.

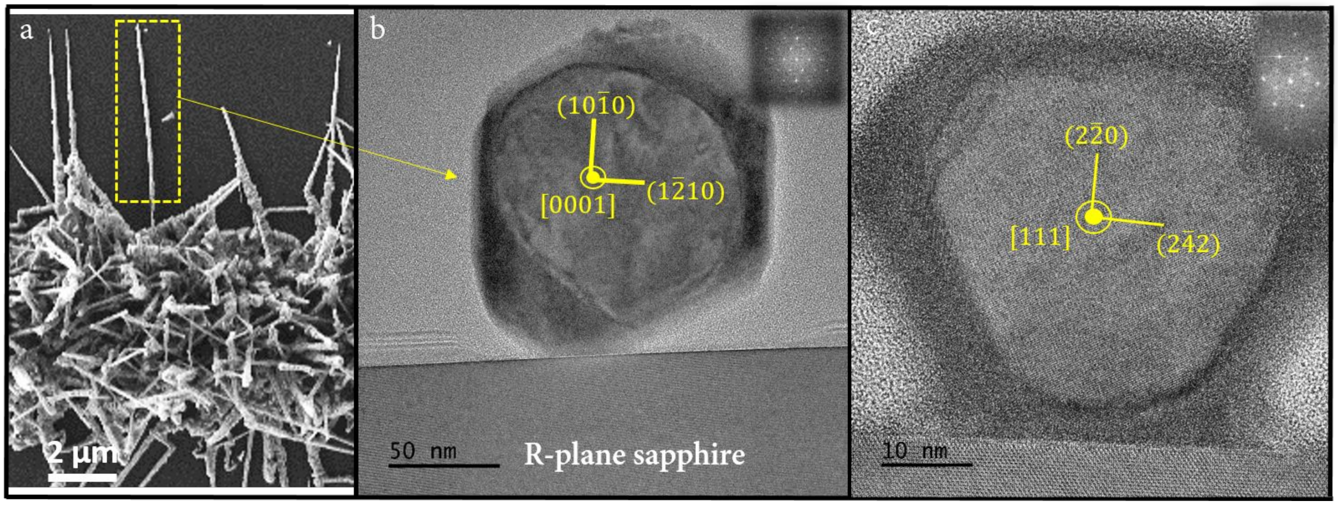

Figure S3. (a) SEM image of a non-planar NW grown on R-plane sapphire (yellow rectangle). (b) Cross-sectional TEM image of a non-planar CdTe NWs. The growth direction and crystal structure are similar to the planar NWs grown on R-plane sapphire [0001] in the WZ structure. (c) Cross-sectional TEM image of another non-planar CdTe NWs showing the parallel direction for ZB symmetry [111]. It strengthens our understanding that the growth direction is dictated not exclusively by the substrate but also by the grown material, which plays a significant role.

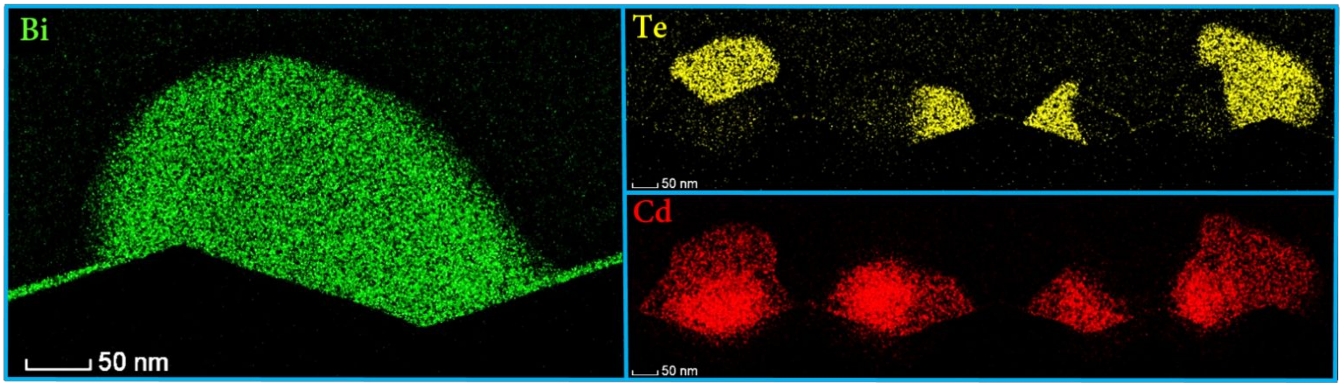

Figure S4. Right, are additional EDS mapping for four wires in a particular lamella cut of NWs on annealed M-plane sapphire. The biphasic structure is clear and is a characteristic of all the examined.

NWs. In the large image to the left, a Bismuth (Bi) map of an image of a NW cross-section in the region of the catalytic droplet. The droplet exhibits a high accumulation of the Bi in it, further, assuring its role as a co-catalyst aside from the gold.

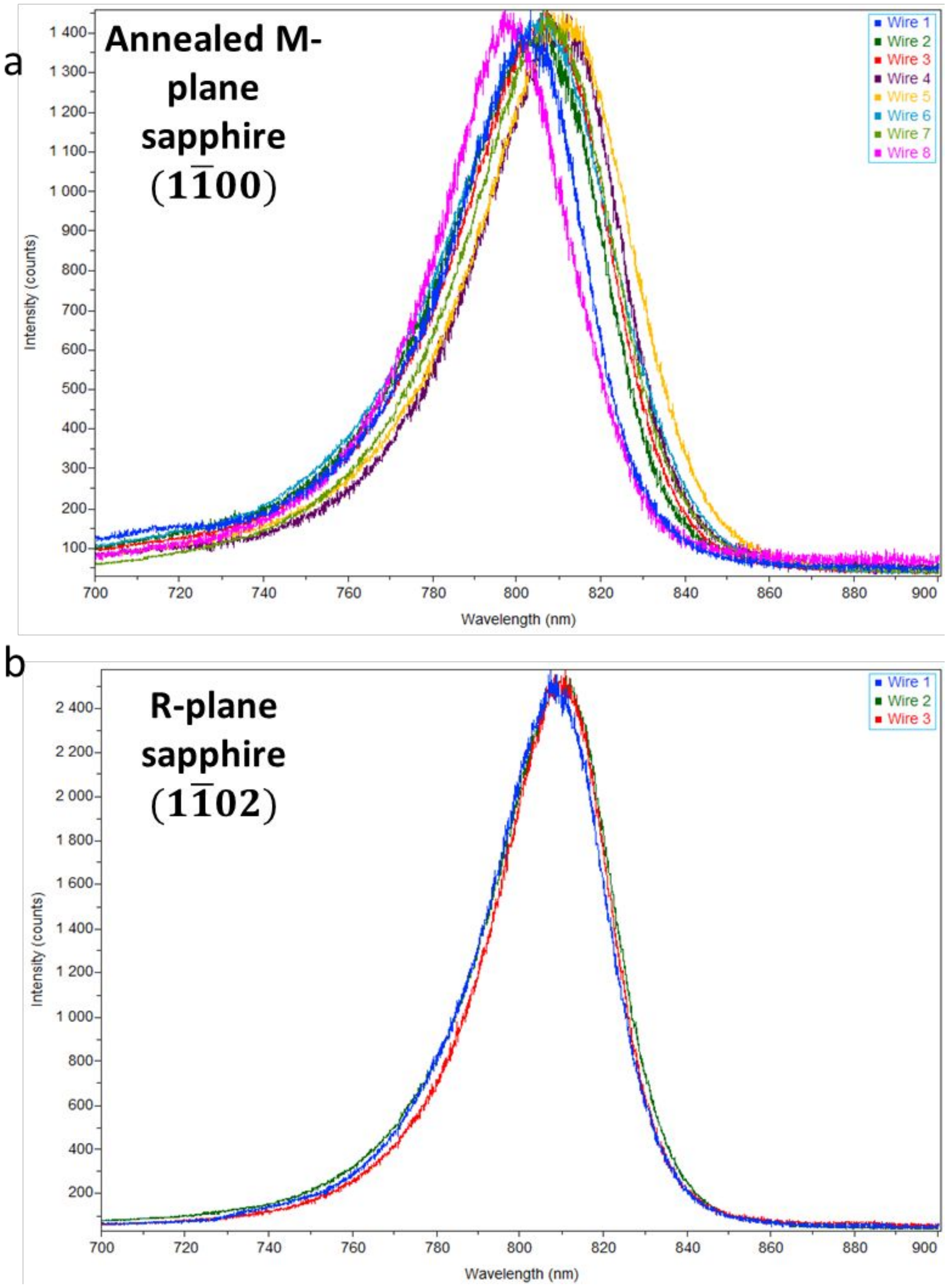

Figure S5. (a) PL spectra were taken from different NWs grown on annealed M-plane $(1\bar{1}00)$ and (b) on R-plane $(1\bar{1}02)$ sapphire, showing deviations from one NW to another. These results correlated with the discussed crystal purity. While the CdTe NWs grown on annealed M-plane sapphire exhibit wide distribution ranges 800-820 nm due to differences in chemical composition, the NWs grown on R-plane sapphire show very narrow distribution with minimal deviations from one NW to another due to higher crystal purity.

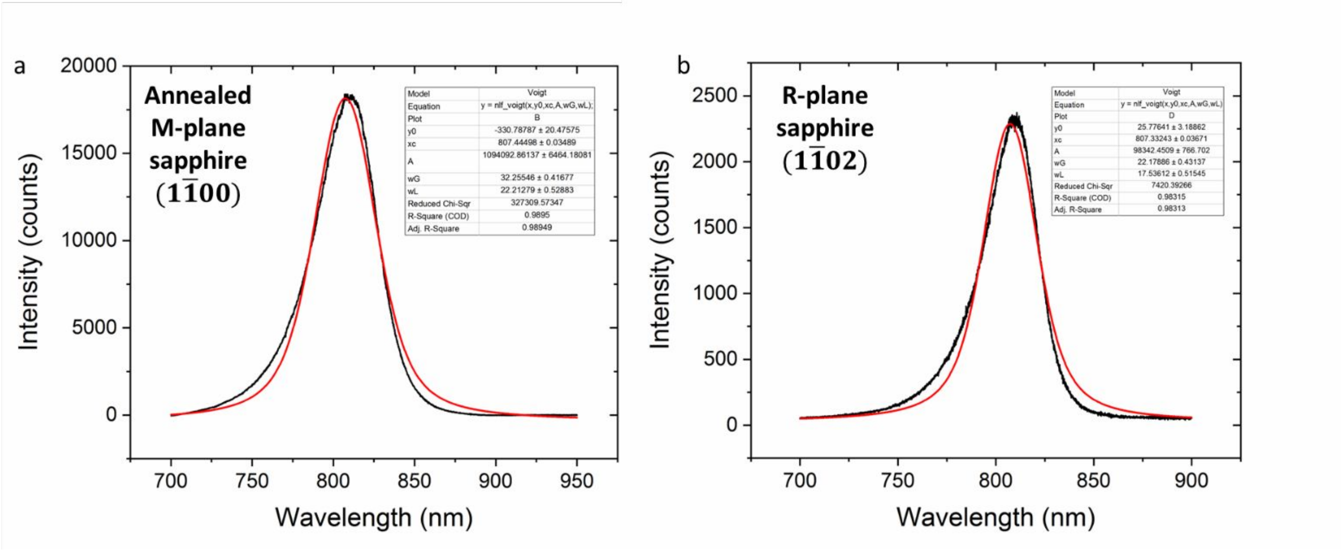

Figure S6. Fitting of the PL spectra collected from single CdTe NW grown on annealed M-plane $(1\bar{1}00)$ (a) and on R-plane $(1\bar{1}02)$ (b) sapphire to a Voigt line shape with the respective fitting parameters reported in the paper. The fitting parameters support the narrower FWHM of the spectra taken from R-plane grown NWs.

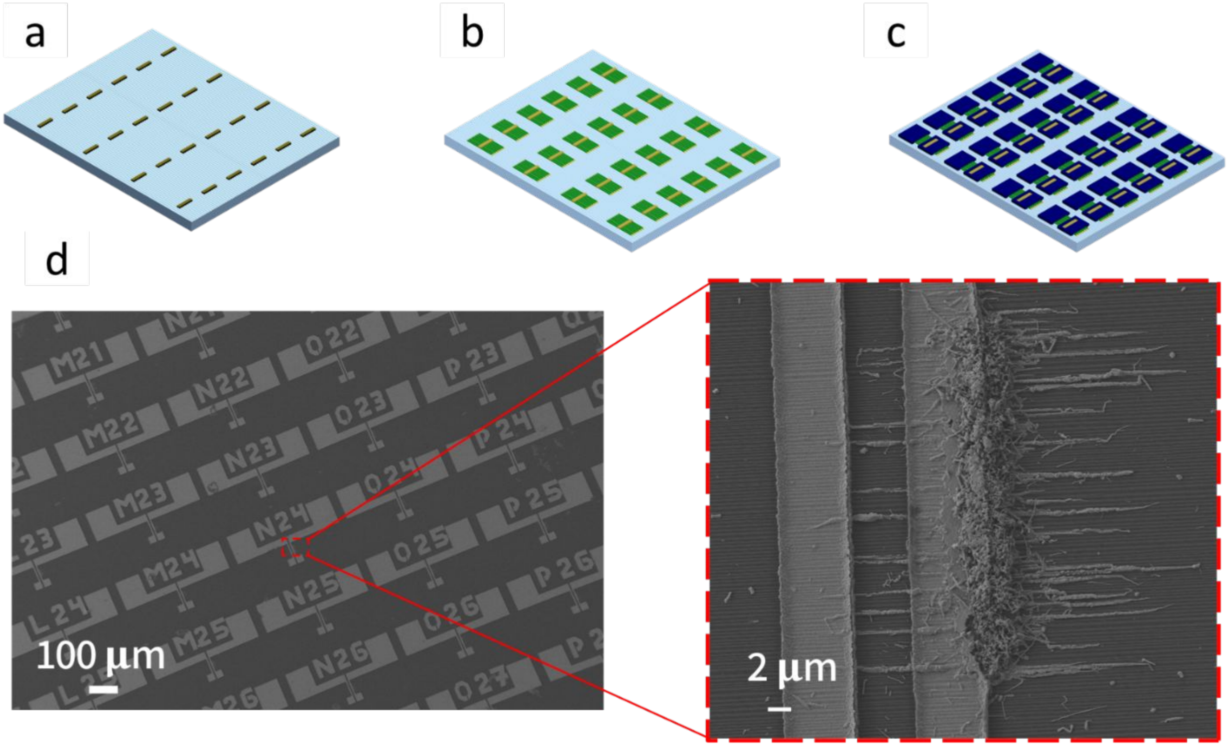

Figure S7. (a) A sketch of gold pads in the size of 3x30 µm that are deposited via lithographic methods in controlled positions on the sapphire substrates prior to the growth process. (b) A sketch of NWs arrays grown from the edges of the lithographic gold pattern. (c) A sketch of photodetectors arrays created based on the previously grown NWs arrays. (d) An SEM image of the photodetectors array, and higher magnification of the channel showing 10 CdTe NWs bridging two gold electrodes.

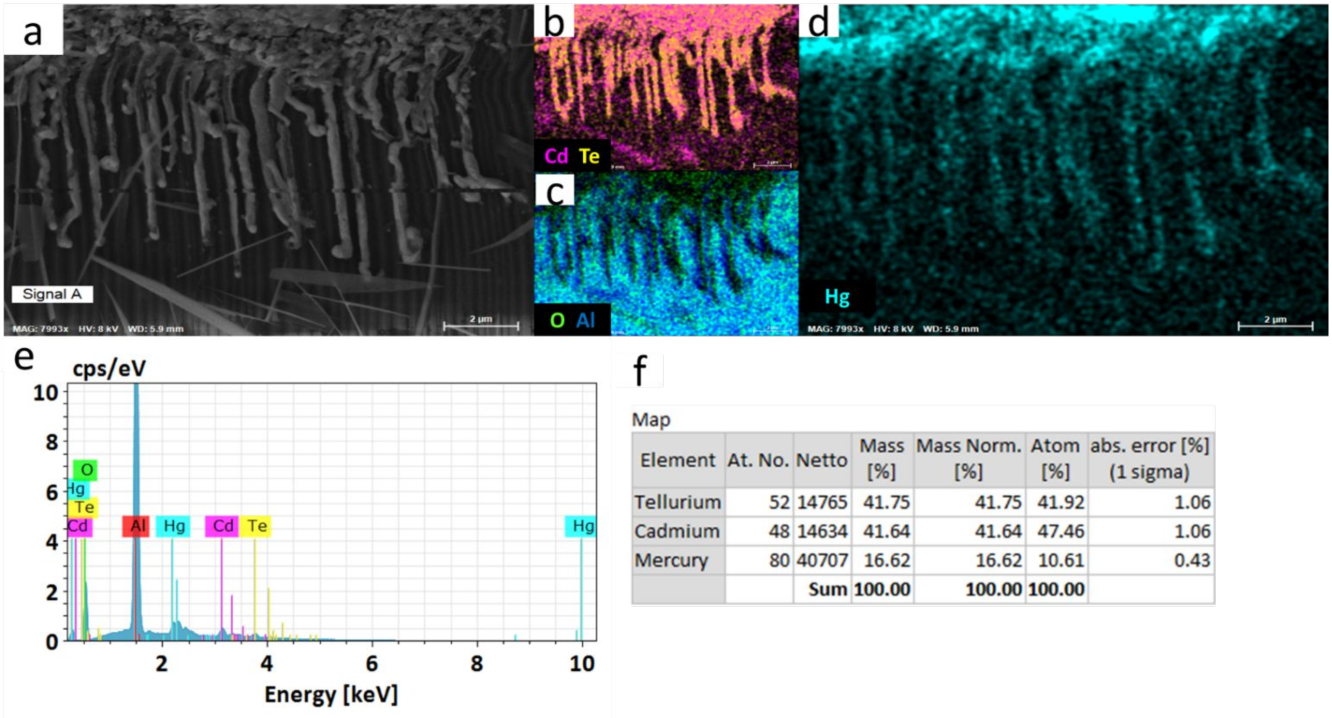

Map

Element	At. No.	Netto	Mass [%]	Mass Norm. [%]	Atom [%]	abs. error [%] (1 sigma)
Tellurium	52	14765	41.75	41.75	41.92	1.06
Cadmium	48	14634	41.64	41.64	47.46	1.06
Mercury	80	40707	16.62	16.62	10.61	0.43
		**Sum**	**100.00**	**100.00**	**100.00**	

Figure S8. (a) A SEM image of CdTe NWs after cation exchange in Hg^{2+} solution. (b) EDS elemental map of typical NWs after cation exchange of Cadmium (magenta) and Tellurium (yellow) and (c) Oxygen (green) and aluminum (light blue) and (d) mercury (cyan) are presented. All scales are 2 µm. (e) The resulting spectrum showing clear signals of mercury, cadmium, and tellurium from the NWs. (f) Quantification of the elements calculated using the spectrum for the mercury, cadmium, and tellurium content in a single NW.

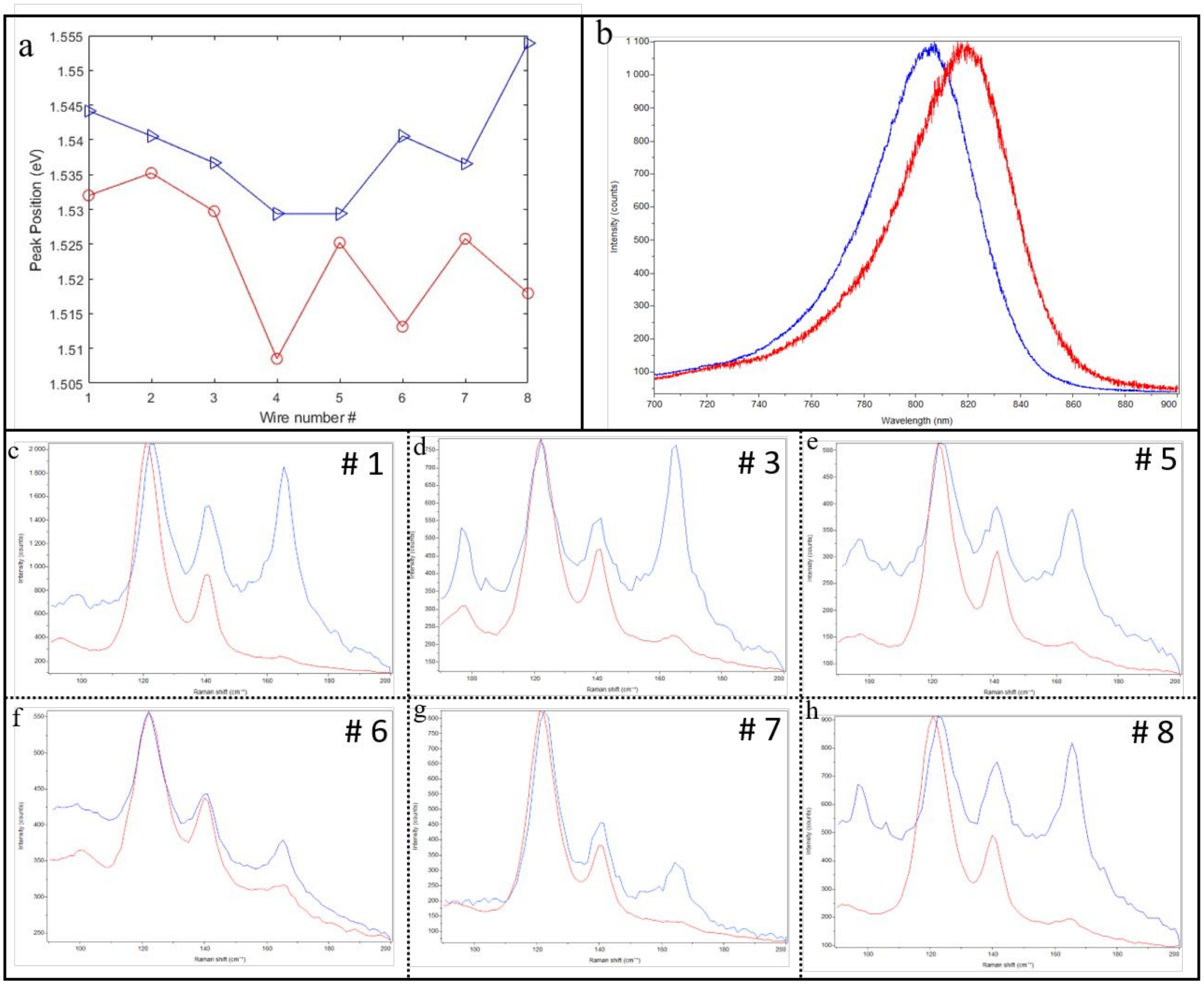

Figure S9. Main results of the cation exchange experiment were done by using $HgCl_2$ dissolved in EtOH solution. (a) The change in near band edge emission by peak position of eight different nanowires went over a cation exchange reaction in $HgCl_2$. Here, a redshift is also observed in all examined wires up to 19 nm, corresponding to 36 meV. (b) A sample spectrum of NW number #8, reported in (a), shows the maximal bandgap narrowing between the starting nanowire (blue) and the exchanged nanowire (red), corresponding 36 meV. (c)-(h) Raman spectra of six different nanowires went over a cation exchange reaction in $HgCl_2$ reported in (a).The apparent decrease in the LO phonon mode intensity is also observed and presented for six different NWs.
